# Uptake of psychotropic medication amongst young adults known to social services in childhood: a longitudinal population-wide cohort study in Northern Ireland.

**DOI:** 10.23889/ijpds.v7i3.1868

**Published:** 2022-08-25

**Authors:** Sarah McKenna, Aideen Maguire, Dermot O'Reilly

**Affiliations:** 1 Queen's University Belfast

## Objectives

To determine the association between childhood involvement with social services and adult mental ill-health, using uptake of psychotropic medication as a proxy indicator.

## Approach

This longitudinal population-wide study linked primary care data on all children born 1985-1991 in Northern Ireland (n=179,484) to social services data (1985-2015) and psychotropic medication prescribing data (2010-2015). Multilevel logistic regression models explored psychotropic medication uptake (antidepressants, anxiolytics, hypnotics and antipsychotics) at ages 18-30 years given exposure to social services in childhood, clustered by GP practice. Additional analysis explored the sociodemographic characteristics and social care experiences (reason known, age at first involvement or entry to care, number of referrals or care episodes and care placement type) associated with medication uptake within social care subgroups.

## Results

Over 1 in 10 adults (10.3%) were known to social services in childhood (3.1% assessed as Not In Need, 5.8% Child In Need, 1.4% In Care). During follow-up 58.5% of care experienced adults were in receipt of psychotropic medication compared to 26.9% of peers never known to social services. Care experienced adults were three to six times more likely to be in receipt of medication (antidepressants OR=3.52, 95% CI 3.25-3.82; antipsychotics OR=6.09, 95% CI 5.37-6.90) than adults never known to social services. Adults deemed Not In Need in childhood (received no services) were at equivalent risk of psychotropic medication to those deemed In Need (received at-home care/support packages). Experiences before and during involvement with social services showed variable association with receipt of psychotropic medication in adulthood.

## Conclusion and Relevance

Adults known to social services in childhood are more likely to receive psychotropic medication than peers never known, with number of referrals and placement type key predictors. Adults known as a Child in Need are equally as likely as those assessed as Not in Need, suggesting possible missed intervention opportunities.

